# Graphene-Scaffolded Ultrathin Perovskite Nanocrystal Films for Amplifying Energy Localization via Dual-Mode Nonhybridizing Quasi-BICs

**DOI:** 10.1021/acs.nanolett.6c00330

**Published:** 2026-03-28

**Authors:** Ya-Lun Ho, Mu-Hsin Chen, Tsung-Hsin Liu, Fong-Liang Hsieh, Chun-Hao Chiang, Chih-Zong Deng, Man-Hong Lai, Jessie Shiue, Shuaicheng Liu, Haruyuki Sakurai, Jui-Han Fu, Kuniaki Konishi, Vincent Tung, Yu-Ming Chang, Chun-Wei Chen, Shao-Ku Huang

**Affiliations:** † Research Center for Electronic and Optical Materials, 52747 National Institute for Materials Science (NIMS), Ibaraki 305-0044, Japan; ‡ International Graduate Program of Molecular Science and Technology (NTU-MST), 33561 National Taiwan University, Taipei 10617, Taiwan; § Molecular Science and Technology Program, Taiwan International Graduate Program (TIGP), Academia Sinica, Taipei 11520, Taiwan; ∥ Department of Materials Science and Engineering, 33561 National Taiwan University, Taipei 10617, Taiwan; ⊥ Center for Condensed Matter Sciences, 33561 National Taiwan University, Taipei 10617, Taiwan; # Institute of Atomic and Molecular Science, Academia Sinica, Taipei 10617, Taiwan; g Institute of Physics, Academia Sinica, Taipei 11520, Taiwan; h Department of Physics, School of Science, 13143 The University of Tokyo, Tokyo 113-0033, Japan; i Institute for Photon Science and Technology, School of Science, 13143 The University of Tokyo, Tokyo 113-0033, Japan; j Department of Chemical System Engineering, School of Engineering, 13143 The University of Tokyo, Tokyo 113-8656, Japan; k Center of Atomic Initiative for New Materials (AI-MAT), 33561 National Taiwan University, Taipei 10617, Taiwan

**Keywords:** Perovskite nanocrystals, Graphene scaffolding, Photonic membrane, Bound states in the continuum (BIC), Dual-mode resonance, Light-matter interaction

## Abstract

Solution-processed metal halide perovskite nanocrystals (NCs) have emerged as exceptional emitters for next-generation optoelectronics and nanophotonics, owing to their high photoluminescence quantum yields and tunable optical properties. However, coupling these colloidal nanomaterials with complex photonic resonators faces severe limitations, particularly on suspended structures where capillary-induced solution leakage disrupts film continuity, fundamentally hindering efficient light–matter interactions. Here, we introduce a graphene-scaffolding strategy that overcomes these limitations, enabling the deterministic fabrication of a continuous, ultrathin (∼20 nm) CsPbBr_3_ NC film on freestanding photonic membranes. The atomically thin graphene interface effectively bridges air holes, preventing nanomaterial leakage and suppressing scattering losses. This architecture provides an ideal nanophotonic platform to exploit engineered dual-mode nonhybridizing bound states in the continuum. By aligning orthogonal resonances for field superposition, we achieve giant energy localization and a record-high (∼200-fold) photoluminescence enhancement. This work highlights 2D-material scaffolding as a universal interface for integrating solution-processed nanomaterials with advanced nanophotonic architectures.

## Graphical abstract

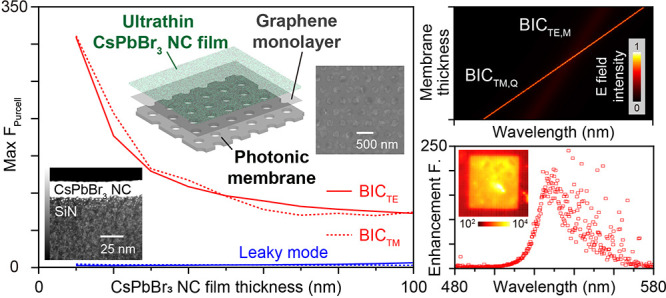

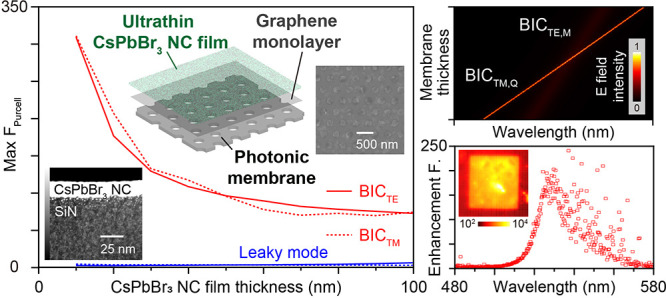

Solution-processed metal halide perovskite (CsPbX_3_, X = Cl, Br, I) nanocrystals (NCs) have emerged as a promising class of nanomaterials for high-performance photovoltaics, optoelectronics, and nanophotonics,^1–13^ owing to their facile synthesis, high absorption efficiency, high photoluminescence quantum yield, and tunable optical bandgaps. Their ability to form scalable thin films via low-cost spin-coating makes them particularly attractive for large-area photonic architectures. However, the critical bottleneck in advancing perovskite nanophotonics lies in effectively coupling these active layers with functional photonic platforms and resonators. Realizing efficient light–matter interaction requires ensuring a maximal spatial overlap between the emitters and the evanescent near-fields of the optical modes.

Achieving such efficient light coupling in the near field uniformly across large-scale device areas presents a persistent challenge arising from the fundamental incompatibility between fluid-phase deposition and nanophotonic topography.^14,15^ Unlike planar substrates, nanophotonic structures possess complex, nonflat geometries required for light confinement. Integrating solution-processed nanomaterials onto these structured platforms introduces substantial fabrication complexities. Conventional spin-coating processes lack the precision to control material assembly at the nanoscale on patterned surfaces; phenomena such as film rupture, discontinuous film formation, and nonuniform solvent evaporation typically lead to uncontrolled material accumulation or discontinuous coverage. Consequently, creating an atomically controllable interface, where an ultrathin and high-quality active layer conforms perfectly to the optical mode volume without degrading the photonic structure, remains a major barrier. This lack of interfacial control leads to suboptimal spatial overlap, severely limiting the potential coupling efficiency and preventing these nanomaterials from fully accessing the enhanced optical fields.

Even beyond these interfacial engineering impediments, selecting an appropriate nanophotonic architecture involves navigating an intrinsic compromise between confinement strength and device scalability. For instance, plasmonic structures offer extreme field confinement but are fundamentally constrained by high ohmic losses and are spatially limited to subwavelength hotspots, hindering their utility for scalable photonic and optoelectronic devices. Conversely, dielectric photonic structures and metasurfaces offer low-loss optical confinement over extended areas. However, conventional designs relying on photonic guided modes typically suffer from weak electromagnetic field confinement compared to plasmonic systems. The optical energy in these systems is often confined primarily within the bulk of the dielectric slab rather than being concentrated at the interface. Consequently, to achieve high field enhancement in these dielectric systems, the precise positioning of the emitter becomes critical. Crucially, because direct ultrathin integration is challenging, thick active layers are frequently employed by default to ensure material continuity. This structural compromise results in poor spatial overlap with the evanescent cavity fields, thereby diluting the effective local field intensity experienced by the emitters and leading to only modest coupling efficiencies.

In this context, bound states in the continuum (BICs) in all-dielectric photonic structures have garnered significant attention as a mechanism to maximize electromagnetic field localization.^16–18^ Theoretically, BICs are localized states of modes that remain nonradiative despite lying within the radiation continuum. In practice, they are realized as quasi-BICs with substantial near-field enhancement, making them highly advantageous for strong excitonic coupling. However, to fully access the strong field enhancement of quasi-BICs, maintaining vertical symmetry is essential, which necessitates the use of suspended or freestanding membranes.^19,20^ Here, the material integration obstacle identified earlier intensifies. Integrating solution-processed perovskites onto porous, freestanding membranes presents a significant challenge. Without a supporting interface, the perovskite NC solution tends to leak through the open air holes, resulting in a fragmented morphology that precludes the formation of the continuous ultrathin film essential for effective near-field coupling.

Here, we introduce a hybrid integration strategy that utilizes monolayer graphene as an atomically thin 2D-material scaffold to interface solution-processed CsPbBr_3_ NCs with freestanding dielectric (SiN) photonic membranes, realizing a giant Purcell enhancement in ultrathin CsPbBr_3_ NC films. The synergistic integration of graphene with halide perovskites has emerged as a promising frontier in optoelectronics, demonstrating exceptional potential in high-performance photovoltaics and functional devices.^21–23^ By leveraging the mechanical robustness of graphene to bridge the air-hole array, we enable the deterministic fabrication of a continuous and ultrathin (∼20 nm) CsPbBr_3_ emissive layer directly on a suspended metasurface—a structural morphology that was previously unattainable via fluid-phase deposition on porous freestanding membranes and substrates. This 2D-material and nanomaterial integration strategy for precise interfacial engineering provides the ideal structural platform to fully exploit advanced nanophotonics. Specifically, we design the membrane to support dual-mode nonhybridizing degenerate quasi-BICs.^24–27^ By precisely tuning the membrane thickness, we align the orthogonal transverse-electric (TE) and transverse-magnetic (TM) quasi-BIC resonances. Because these modes do not hybridize, their electromagnetic fields constructively superpose, substantially enhancing the available local density of optical states and creating a strongly localized field at the membrane surface. Our ultrathin, graphene-scaffolded NC film resolves the critical trade-off between nanomaterial continuity and spatial overlap, ensuring that the emitters are confined precisely within this enhanced field region. Consequently, we experimentally demonstrate a record-high photoluminescence (PL) enhancement exceeding 200-fold, significantly surpassing conventional dielectric and plasmonic architectures for perovskite NC thin films.^28–38^ Beyond this specific demonstration, our work highlights the utility of 2D-material scaffolding as a universal interface, offering a robust and scalable pathway for integrating diverse solution-processed nanomaterials with complex nanophotonic structures.

To realize efficient coupling with the photonic membrane architecture, the fabrication of a high-quality, continuous, and ultrathin emissive layer serves as an essential building block. We first synthesized all-inorganic CsPbBr_3_ NCs^39,40^ and integrated them onto the monolayer graphene (see Supporting Information and Figure S1). The structural integrity of the synthesized NCs was confirmed by X-ray diffraction (XRD). As shown in Figure 1a, the diffraction peaks correspond well to the standard cubic perovskite structure, indicating high crystallinity and phase purity. The optical quality is further evidenced by the PL spectrum (Figure 1b), which exhibits a sharp, symmetric emission peak centered at ∼518 nm with a narrow line width, characteristic of high-quality excitonic emission with minimal defect-induced broadening. Precise control over the film thickness is essential to maximize the spatial overlap between the CsPbBr_3_ NC thin film and the highly confined electromagnetic fields of the quasi-BIC modes. We systematically optimized the film deposition process by varying the spin-coating speed. Figure 1c presents the cross-sectional SEM images of the CsPbBr_3_ layers fabricated at 2000, 6000, and 8000 rpm. A monotonic reduction in film thickness is observed as the rotational speed increases. The thickness decreases from ∼94 nm at 2000 rpm to ∼52 nm at 6000 rpm, and finally to an ultrathin dimension of ∼28 nm at 8000 rpm. Crucially, despite the ultrathin nature of the film deposited at 8000 rpm, the layer maintains excellent continuity and surface uniformity. The atomic force microscopy (AFM) topography image (Figure 1c, right) reveals a dense and homogeneous morphology with a remarkably low root-mean-square roughness R_a_ = 1.4 nm. Achieving such a smooth and continuous ultrathin film is crucial for our device architecture. It maximizes the spatial overlap between the CsPbBr_3_ NCs and the intense evanescent near-fields of the photonic membrane, thereby facilitating the efficient light-matter interaction required for the giant PL amplification discussed in subsequent sections.

**Figure 1:**
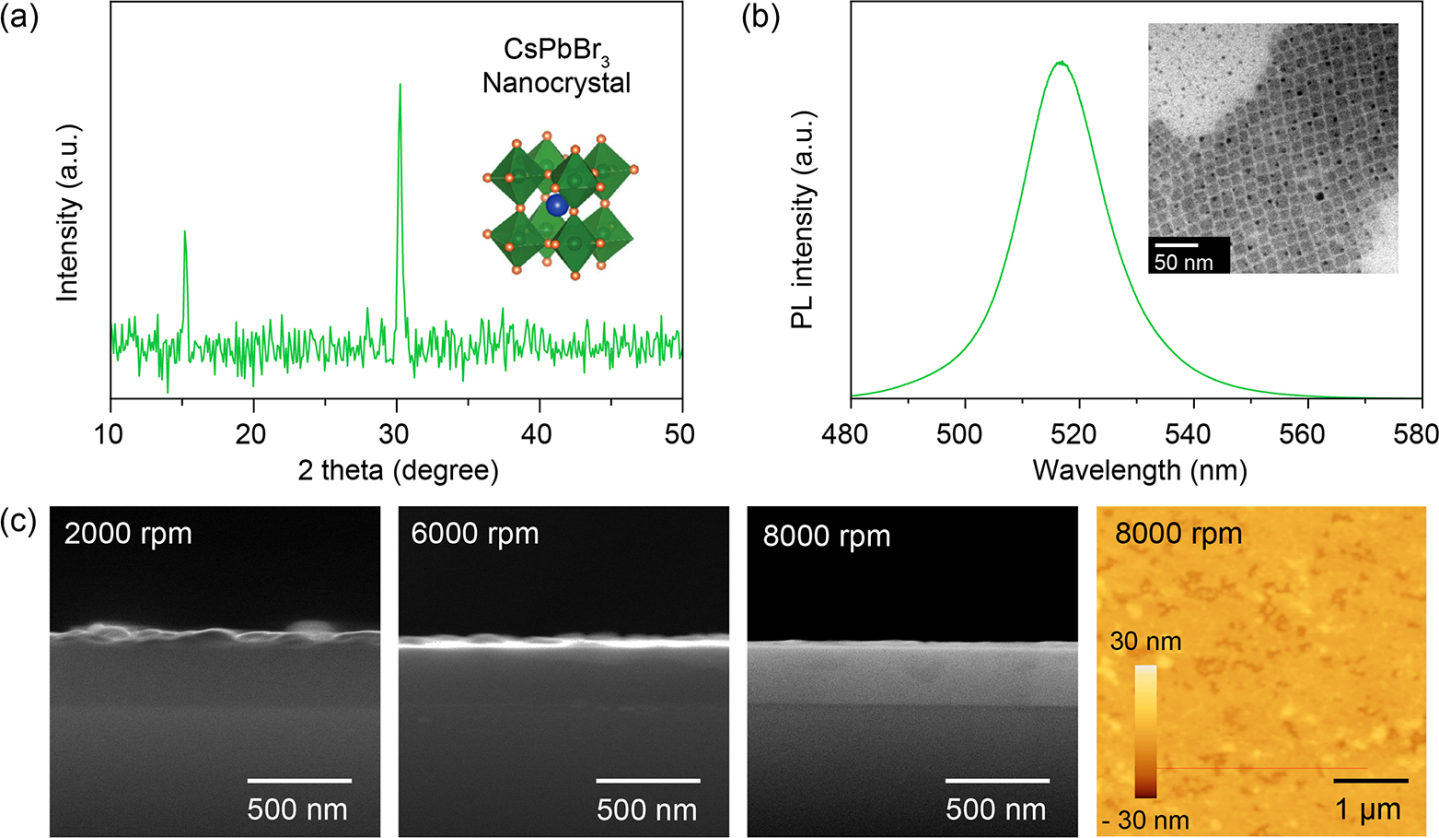
Characterization of the CsPbBr_3_ NC thin film. (a) X-ray diffraction (XRD) pattern of the synthesized CsPbBr_3_ nanocrystals (NCs), confirming the high crystallinity of the perovskite structure (inset shows the unit cell schematic). (b) Photoluminescence (PL) spectrum of the CsPbBr_3_ NC thin film, showing a narrow emission bandwidth typical of high-quality perovskite NCs. The inset displays a transmission electron microscopy (TEM) image revealing well-defined cubic morphology with an average edge length consistent with high-quality CsPbBr_3_ NCs. (c) Cross-sectional scanning electron microscopy (SEM) images of the CsPbBr_3_ films deposited on SiO_2_/Si substrates at spin speeds of 2000, 6000, and 8000 rpm, respectively (scale bar: 500 nm). The images reveal a clear reduction in film thickness with increasing spin speed. The corresponding atomic force microscopy (AFM) topography image (right) of the film prepared at 8000 rpm demonstrates a smooth and uniform surface morphology (scale bar: 1 μm).

While the optimized spin-coating parameters yield high-quality NC films on solid substrates, integrating them with hole-array photonic membranes presents a significant challenge. Direct deposition typically leads to uncontrolled solution leakage, where the NC solution flows through the air holes rather than forming a continuous film. To overcome this, we introduced a monolayer graphene sheet as an atomically thin, mechanical scaffold to support the CsPbBr_3_ NCs. Figure 2a illustrates the schematic of this hybrid architecture, where the graphene acts as an impermeable interface between the colloidal CsPbBr_3_ NCs and the SiN photonic membrane.

**Figure 2:**
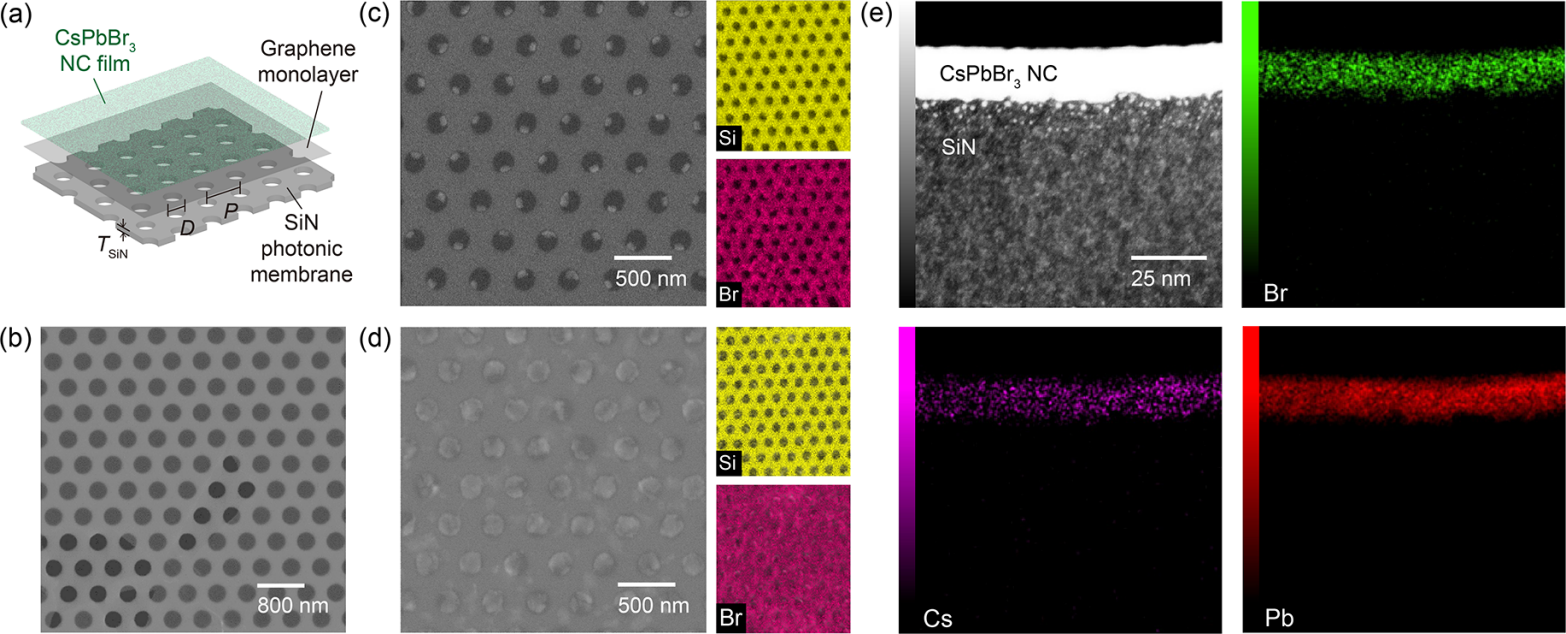
Graphene-scaffolded hybrid photonic architecture integrating CsPbBr_3_ NC thin films. (a) Schematic illustration of the ultrathin CsPbBr_3_ NC film integrated with a dielectric photonic membrane, highlighting the monolayer graphene as a structural scaffold. Geometric parameters *T*_SiN_, *D*, and *P* indicate the thickness, hole diameter, and period of the SiN dielectric membrane, respectively. (b) Top-view SEM image of the monolayer graphene transferred onto the photonic membrane. A partially ruptured area is intentionally imaged to provide contrast, confirming the freestanding nature of graphene over the air holes (scale bar: 800 nm). (c, d) SEM images comparing CsPbBr_3_ NCs spin-coated onto the photonic membrane (c) without and (d) with the graphene scaffold (scale bar: 500 nm). The corresponding energy-dispersive X-ray spectroscopy (EDS) elemental maps for Si and Br are shown alongside. (e) Cross-sectional scanning transmission electron microscopy (STEM) image of the CsPbBr_3_ NC film on the photonic membrane, showing a uniform thickness of ∼20 nm. Corresponding EDS maps confirm the chemical homogeneity.

The structural integrity of the transferred graphene is verified in Figure 2b, where a partially ruptured area is intentionally imaged to provide contrast, confirming that the graphene is successfully suspended over the air holes without sagging or tearing. Additional SEM images demonstrating the large-area uniformity and defect-free nature of the graphene are provided in Figure S2. The critical role of this graphene interface is highlighted by comparing the morphology of CsPbBr_3_ NCs deposited in the presence and absence of the graphene scaffold. In the absence of graphene (Figure 2c), the NC solution predominantly leaks through the holes due to surface tension effects, resulting in a discontinuous morphology. This discontinuity drastically reduces the effective active area and spatial overlap with the photonic modes, rendering the light-matter interaction inefficient. Furthermore, the irregular accumulation at the hole edges introduces severe scattering losses, which further degrade the optical performance. In contrast, the graphene-supported architecture (Figure 2d) enables the formation of a continuous CsPbBr_3_ NC thin film that floats over the photonic membrane. The film maintains the ultrathin thickness achieved on solid substrates, effectively bridging the air holes. This structural uniformity is further corroborated by energy-dispersive X-ray spectroscopy (EDS) mapping. Without graphene, the Br signal (magenta) is concentrated within the dielectric surface (Figure 2c), and the absence of signal in the holes indicates solution drainage. With the graphene support, the Br signal becomes spatially homogeneous (Figure 2d), confirming that the NCs are uniformly distributed across the entire surface. To verify the NC thin film thickness on the photonic membrane, cross-sectional scanning transmission electron microscopy (STEM) analysis was performed (Figure 2e). The STEM image clearly reveals the NC layer with a thickness of approximately 20 nm directly spanning the SiN membrane, fully consistent with the morphology observed on planar substrates. The corresponding EDS elemental mapping further confirms the spatial homogeneity within the suspended film, validating the robustness of our graphene-scaffolding strategy. This architecture offers several decisive advantages for nanophotonic applications. First, it significantly maximizes the effective active area by preventing material loss into the membrane voids. Second, the atomically flat nature of graphene prevents the formation of edge-induced thickness variations at the hole boundaries, thereby suppressing scattering losses. Consequently, this design realizes a truly freestanding active layer suspended over the air-hole array, providing a structurally robust yet optically accessible platform essential for the excitation of high-Q resonances. Beyond its structural role, we also evaluated the potential optical influence of the graphene interface. Control PL measurements performed on planar substrates (Figure S3) reveal that the presence of the graphene monolayer results in only a minor reduction in emission intensity (∼7%) compared to a pristine substrate. This confirms that graphene acts primarily as a transparent mechanical scaffold without inducing significant nonradiative quenching or absorption losses, thereby preserving the high emission efficiency of the perovskite NCs.

To elucidate the light-matter coupling mechanisms in the ultrathin graphene-CsPbBr_3_ heterostructure, we first designed and analyzed the freestanding dielectric (SiN) photonic membrane as the nanophotonic platform. The photonic membrane architecture consists of a freestanding SiN slab patterned with a hexagonal lattice with *C*_6_ symmetry of air holes, as illustrated in Figure 2a and the inset of Figure 3a. The quasi-BIC resonances in such photonic membrane are highly sensitive to structural parameters, specifically lattice period (*P*), air-hole diameter (*D*), and membrane thickness (*T*_SiN_), which provide precise control over the resonance wavelength and electromagnetic field distribution. In this study, the lattice period (*P* = 400 nm) and hole diameter (*D* = 200 nm) were optimized to position the quasi-BIC resonances within the emission band of the CsPbBr_3_ NCs, ensuring spectral overlap while maintaining fabrication feasibility. Subsequently, the membrane thickness was leveraged as a fine-tuning parameter to precisely align the cavity modes with the excitonic transition.

**Figure 3:**
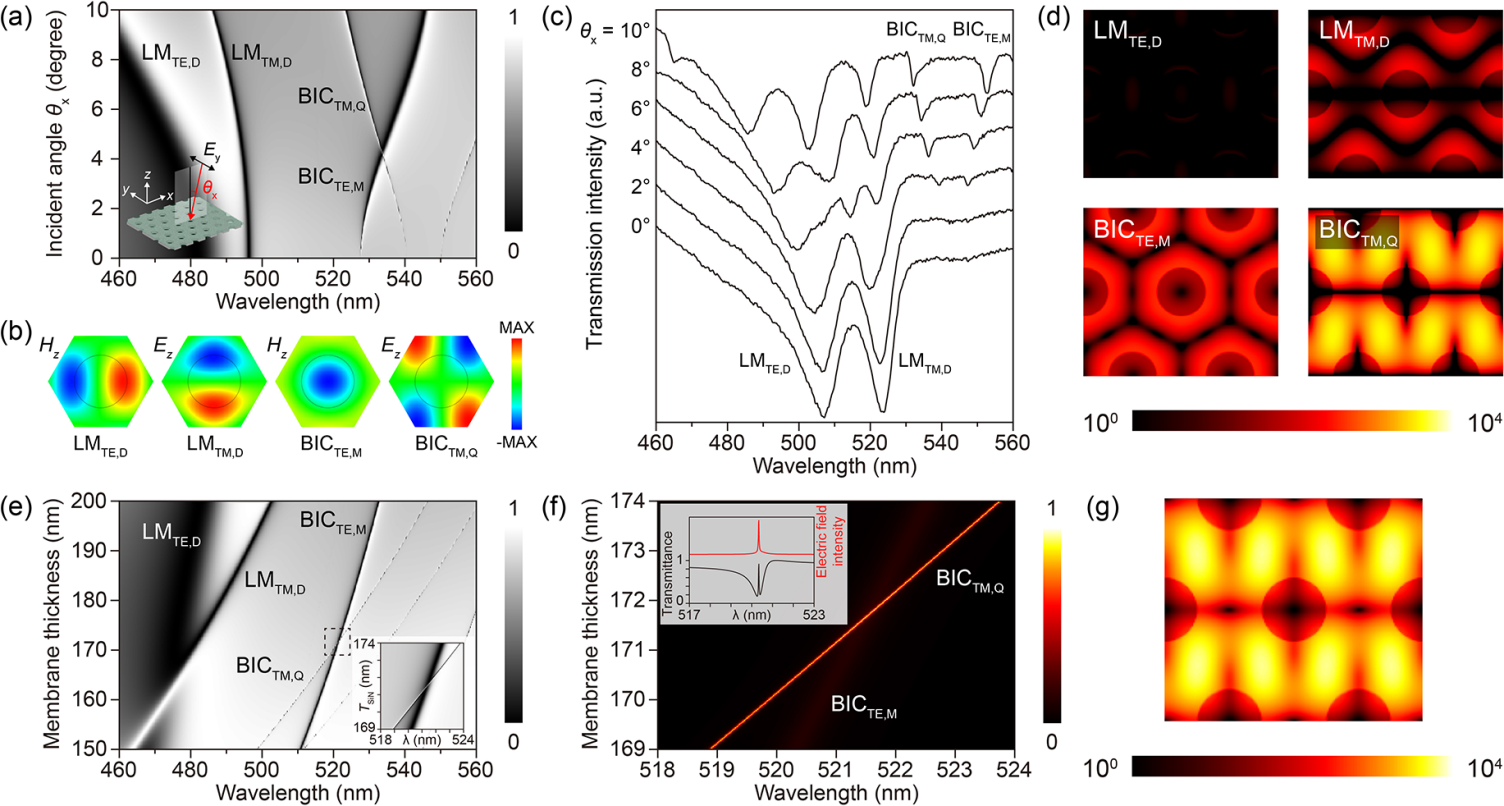
Dual-mode nonhybridizing degenerate quasi-BICs for enhanced energy localization. (a) Simulated angle-resolved transmittance spectrum for a photonic membrane with a thickness *T*_SiN_ = 190 nm. The dispersion map distinguishes the leaky modes (LMs) from the quasi-bound states in the continuum (BICs) with the characteristic vanishing of the BIC at the Γ point. (b) Simulated out-of-plane field distributions (*H*_*z*_ or *E*_*z*_) of the resonances indicated in (a), revealing their TE- or TM-like multipolar field configurations, including the dipole LMs (LM_TE,D_ and LM_TM,D_), the monopole quasi-BIC (BIC_TE,M_), and the quadrupole quasi-BIC (BIC_TM,Q_). (c) Measured angle-resolved transmittance spectra. (d) Electric energy density distributions of the LMs and BICs at an incident angle θ_*x*_ = 2°, shown at the center plane of the membrane. The color scale is logarithmic. (e) Simulated spectra as a function of membrane thickness *T* at a fixed θ_*x*_ = 2°. The map reveals the tunability of mode positions and the intersection of BIC_TE,M_ and BIC_TM,Q_ (Inset). (f) Spectral mapping of the normalized spatially integrated electric field intensity. The BIC_TE,M_ and BIC_TM,Q_ cross without anticrossing behavior, confirming their nonhybridizing nature. The inset displays the simulated transmittance spectrum (black) and electric field intensity (red) at *T*_SiN_ = 171.5 nm, indicating maximal field enhancement at the degenerate point. (g) Electric energy density distributions corresponding to the degenerate quasi-BICs in (f), demonstrating the simultaneous excitation of TE- and TM-like characteristics with enhanced field distribution at the center plane.

The optical properties were simulated using rigorous coupled-wave analysis (RCWA) method (DiffractMOD, RSoft Design Group, USA). Simulation details are provided in the Supporting Information. Figure 3a presents the simulated angle-resolved transmittance spectrum under x-polarized excitation, mapped by varying the incident angle θ_*x*_ along the *x*-direction, for a membrane thickness of *T*_SiN_ = 190 nm. It should be noted that at off-normal incidence, the broken symmetry allows the linearly polarized light to couple to both TE-like and TM-like modes. The dispersion map reveals two distinct classes of modes: the spectrally broad leaky modes (LMs) in the shorter wavelength range (<500 nm) and the narrow-line width quasi-BICs in the longer wavelength range (>520 nm). A defining feature of the quasi-BICs is the vanishing of the resonance line width at the Γ point (normal incidence), confirming their symmetry-protected nature where radiation channels are suppressed. To elucidate the nature of these modes, we verified their multipolar characteristics governed by the *C*_6_ symmetry of the photonic lattice by analyzing the out-of-plane field components (*H*_*z*_ or *E*_*z*_) in Figure 3b. Specifically, the resonances in Figure 3a are identified as leaky modes with TE and TM dipole characteristics (denoted as LM_TE,D_ and LM_TM,D_), alongside BICs featuring TE monopole and TM quadrupole symmetries (BIC_TE,M_ and BIC_TM,Q_). Furthermore, these modes were experimentally verified through transmittance measurements in Figure 3c, which exhibit good agreement with the numerical simulations (see Supporting Information, Figure S4 for detailed discussion). Subsequently, we analyzed their electric energy density distributions at a small incident angle of θ_*x*_ = 2° (Figure 3d). The LMs exhibit relatively low energy confinement, with field enhancements on the order of 10.^2^ In contrast, the BIC_TE,M_ and BIC_TM,Q_ modes demonstrate significantly higher energy densities, reaching orders of 10^3^ to 10^4^. Spatially, the high-energy fields of the quasi-BICs are localized at the boundaries of the air holes and within the dielectric connecting regions, providing an ideal environment for enhancing light-matter interaction with the overlying NC film. In addition, to provide a complete mode analysis of the guided resonances governed by the *C*_6_ symmetry of the hexagonal lattice, we calculated the full *E*-*k* band diagrams for both TE and TM polarizations (Figure S5). These diagrams comprehensively map the modal dispersion and identify all characteristic multipole modes (monopole, dipole, quadrupole, and hexapole) supported by the system.

The pivotal innovation of this design lies in the precise manipulation of modal interaction via thickness tuning. Figure 3e illustrates the evolution of the simulated spectra as a function of membrane thickness at θ_*x*_ = 2°. As *T* decreases, the resonance wavelengths blueshift due to the reduction in the effective refractive index. Crucially, because the TE-like and TM-like modes possess different field confinement profiles perpendicular to the slab, their spectral sensitivities to thickness differ. This allows the BIC_TE,M_ and BIC_TM,Q_ branches to intersect at a specific thickness (The inset of Figure 3e). Detailed spectral mapping of the normalized spatially integrated electric field intensity (Figure 3f) reveals the formation of a degeneracy point at this intersection. Unlike typical coupled-mode systems that exhibit anticrossing behavior (Rabi splitting) due to mode hybridization, the BIC_TE,M_ and BIC_TM,Q_ branches cross each other without opening a spectral gap. This behavior confirms that the two modes possess orthogonal symmetries and do not hybridize. However, while their eigenstates remain orthogonal, their electromagnetic energies constructively superpose. At the degenerate condition (*T* ≈ 171.5 nm), the internal field intensity reaches a global maximum (Figure 3f, Inset). The corresponding electric energy density distribution (Figure 3g) displays simultaneous TE-like and TM-like characteristics with high spatial overlap. This dual-mode nonhybridizing degeneracy effectively amplifies the local density of optical states available for the emitter, providing a robust pathway for maximizing PL enhancement.

Having established the existence of strongly enhanced field localization from quasi-BICs, we proceeded to quantify the coupling strength between the CsPbBr_3_ excitons and the photonic modes. We performed three-dimensional finite-element method (FEM) simulations using COMSOL Multiphysics (COMSOL, Inc., USA). In these calculations, the photonic membrane thickness was set to 160 nm to align with the optimized experimental resonance condition, and the CsPbBr_3_ NC film was modeled as a dispersive gain medium on top of the graphene interface. Figure 4a displays the calculated maximum Purcell factor (F_Purcell_) as a function of the CsPbBr_3_ NC film thickness (*T*_CsPbBr3_). A striking inverse dependence is observed: while the LMs provide limited enhancement (*F*_Purcell_ < 5) regardless of thickness, the quasi-BICs exhibit a dramatic rise in F_Purcell_ as the film thickness enters the regime smaller than 30 nm. Specifically, as the film thickness decreases from 100 to 10 nm, the *F*_Purcell_ for the BIC_TE,M_ and BIC_TM,Q_ surges to approximately 300. This value represents a state-of-the-art enhancement for solution-processed perovskite metasurfaces,^28,31,32,36,38^ comparable to or exceeding values typically achieved in highly confined plasmonic systems,^41,42^ but without the associated ohmic losses. To elucidate the physical origin of this giant enhancement, we analyzed both the cross-sectional Purcell factor distributions and the in-plane spatial distributions calculated at the center plane of the NC film in Figure 4b–e. The cross-sectional distributions reveal that the quasi-BIC mode energy is strongly localized at the membrane interface due to the divergence of the local density of states. Consequently, the ultrathin (∼20 nm) NC film geometry is critical, as it confines the emitters exclusively within this near-field region, allowing them to access the maximum enhancement. In contrast, a thicker film would extend into regions of lower field intensity, leading to a reduced average enhancement due to volume averaging effects. This depth-dependent field decay is visually confirmed by comparing the in-plane spatial distribution maps calculated at the center plane of the NC film. For a relatively thick film (*T*_CsPbBr3_ = 80 nm, Figure 4b,d), the calculated enhancement is moderate because the center plane is distant from the photonic membrane surface with strong field enhancement. However, for the ultrathin film (*T*_CsPbBr3_ = 20 nm, Figure 4c,e), the center plane coincides with the region of maximum field localization, allowing the emitters to fully exploit the intense electromagnetic environment of the degenerate quasi-BICs. These results rigorously validate our fabrication strategy, confirming that achieving an ultrathin, continuous film is not merely a structural preference but a fundamental optical requirement to unlock the giant Purcell enhancement provided by the degenerate quasi-BICs.

**Figure 4:**
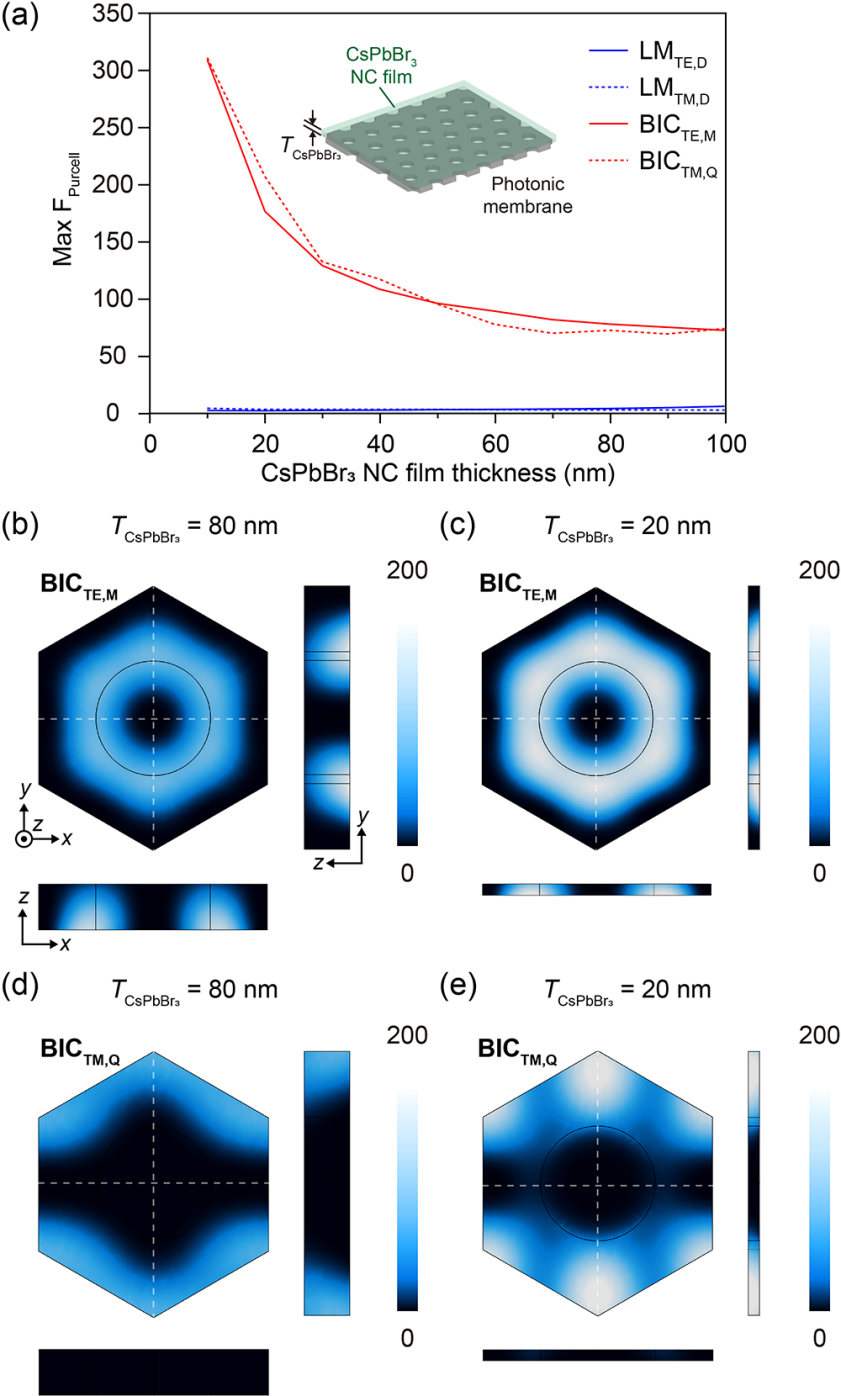
Purcell enhancement in the ultrathin graphene-scaffolded CsPbBr_3_ NC heterostructure. (a) Simulated maximum Purcell factor (*F*_Purcell_) variation as a function of the CsPbBr_3_ NC film thickness (*T*_CsPbBr3_). The values are extracted from the center plane of the emitting layer. The plot reveals a pronounced enhancement in *F*_Purcell_ for both BIC_TE,M_ and BIC_TM,Q_ (red lines) as the film thickness decreases into the ultrathin regime (<30 nm), exhibiting orders-of-magnitude higher values than the LMs (blue lines). The inset illustrates the simulation geometry. (b–e) Spatial distributions of the Purcell factor calculated at the center plane of the CsPbBr_3_ NC film, along with the corresponding cross-sectional distributions, for the BIC_TE,M_ (b, c) and BIC_TM,Q_ (d, e). The images compare the thick film case (*T*_CsPbBr3_ = 80 nm) shown in (b, d) with the ultrathin film case (*T*_CsPbBr3_ = 20 nm) shown in (c, e).

To experimentally validate the proposed design, we first investigated the impact of the film continuity preserved by the graphene scaffold on optical confinement. The measurements were conducted using a 405 nm continuous-wave laser focused by a 10× objective lens (NA = 0.25) to a diffraction-limited spot. The excitation power density was maintained at 150 W/cm^2^ to prevent thermal degradation. To ensure that the observed giant enhancement is not an artifact of saturation, we verified the linear dependence of the PL intensity with respect to the excitation power (Figure S6). This linearity confirms the absence of saturation or photobleaching effects under our measurement conditions. Figure 5a and b presents the PL spectra of CsPbBr3 NC films integrated on photonic membranes (*T*_SiN_ = 190 nm) without and with the monolayer graphene interface, respectively. While both samples exhibit PL modulation due to the LMs, the modal distribution reveals an important distinction. As shown in the insets, the discontinuous, infiltrated morphology in the graphene-free sample results in a relatively low confinement factor (C.F.) of 1.62%. In contrast, the continuous, graphene-supported ultrathin film significantly boosts the C.F. to 3.54%, representing a more than 2-fold increase. This enhancement confirms that the freestanding nature of the film, enabled by the graphene layer, is indispensable for maximizing the overlap between the active medium and the optical modes. This improved optical confinement directly translates into a substantial enhancement in PL intensity. The cross-sectional electric energy density distributions and the calculated C.F. values for all other investigated modes, including both the leaky modes and the quasi-BICs, are provided in Figure S7.

**Figure 5:**
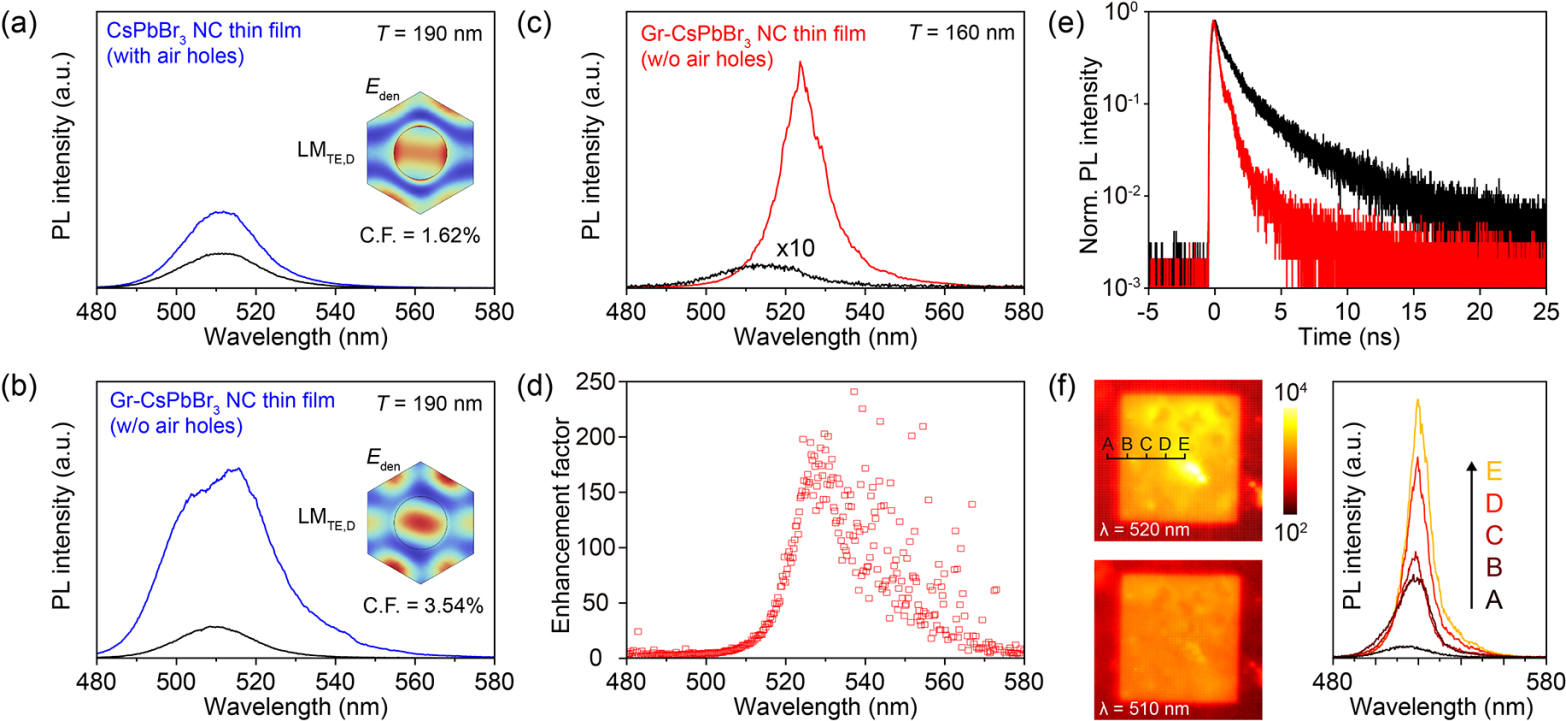
Luminescence enhancement in ultrathin graphene-scaffolded CsPbBr_3_ NC heterostructures mediated by degenerate quasi-BICs. PL spectra of (a) the CsPbBr_3_ NC film (w/o graphene) and (b) the graphene-scaffolded CsPbBr_3_ NC film on the photonic membrane (*T*_SiN_ = 190 nm, blue lines), compared to the reference spectra on unstructured membranes (black lines). The insets display the electric energy density distributions and the calculated confinement factors (C.F.), indicating that the graphene-scaffolded continuous NC film achieves a significantly higher C.F. (3.54% vs 1.62%). (c) PL spectra of the graphene-scaffolded CsPbBr_3_ NC film at the optimized membrane thickness (*T*_SiN_ = 160 nm) for dual-mode degenerate quasi-BICs. The excitation of the quasi-BIC resonance results in a substantial increase in emission intensity (red line) relative to the reference (black line). (d) Corresponding enhancement factor spectrum extracted from (c), revealing a peak enhancement factor exceeding 200-fold. (e) Time-resolved PL decay curves of CsPbBr_3_ NC film coupled to the quasi-BIC resonance (red), compared to reference NCs on the unstructured membrane (black). (f) Spatially resolved PL intensity mapping of the graphene-scaffolded CsPbBr3 NC film on the photonic membrane at the resonance wavelength (λ = 520 nm, top) and at the intrinsic emission peak (λ = 510 nm, bottom). The right panel shows the evolution of PL spectra collected from positions A to E.

Building on this optimized integration, we fine-tuned the membrane thickness to the theoretical optimum of *T*_SiN_ = 160 nm to access the regime of dual-mode nonhybridizing degenerate quasi-BICs. Figure 5c shows the PL spectrum under this condition. A pronounced enhancement in emission intensity is observed at the resonance wavelength, significantly surpassing the limited enhancement attributed to LMs. The corresponding enhancement factor spectrum (Figure 5d) reveals a peak value exceeding 200-fold. To quantify this enhancement, we performed PL measurements comparing the emission from the photonic-structure (hole array) region to that from the adjacent unstructured membrane on the same sample. It is worth noting that the PL enhancement in photonic structures can be expressed as *I*_PL_ ∝ η_abs_ × η_rad_ × η_out_, where η_abs_ is absorption, η_rad_ is radiative efficiency (governed by the radiative rate), and η_out_ is light extraction efficiency. The observed PL enhancement originates from a synergistic combination of two physical mechanisms, specifically the increased light extraction efficiency due to the directional out-coupling of the photonic crystal mode at the Γ-point and the enhanced radiative decay rate driven by the Purcell effect. To verify the presence of the Purcell effect, time-resolved PL measurements were performed (Figure 5e). The PL decay curves show a significant reduction in lifetime for the NCs coupled to the quasi-BIC mode (τ ≈ 0.8 ns) compared to the reference NCs on the unstructured membrane (τ ≈ 2.2 ns). This accelerated decay confirms that the photonic membrane effectively modifies the local density of optical states, leading to a faster radiative rate consistent with the Purcell enhancement mechanism.

Furthermore, regarding the quantification of the enhancement factor, we note that the resonance-specific amplification reaches ∼200-fold. This metric physically quantifies the amplification capability of the resonant mode itself. The observed spectral shift between the enhanced peak and the intrinsic NC emission peak likely arises from a slight spectral detuning between the fabricated structural resonance and the NC emission center. Thus, comparing intensities at the resonance wavelength effectively isolates the photonic resonance-induced amplification. However, for practical device performance, comparing the global peak intensities is equally important. We therefore calculated the peak-to-peak enhancement factor, which yields a value of ∼106-fold. Both metrics confirm that the graphene-scaffolded ultrathin perovskite NC film architecture provides a giant amplification of light emission.

To benchmark this performance, we compared our result with recent reports on perovskite NC luminescence enhancement in various nanophotonic architectures. It is crucial to distinguish between localized enhancement in single-particle configurations and area-scalable enhancement in continuous thin films. While isolated NCs coupled to plasmonic hotspots can exhibit high local field intensities,^41–43^ they are inherently limited to microscopic volumes and suffer from ohmic losses. For practical optoelectronic applications requiring spatially extended emission, achieving high enhancement in continuous thin films is significantly more challenging due to the trade-off between mode overlap and film volume. Most reported enhancement factors for perovskite films integrated with dielectric nanoresonators, metasurfaces, and photonic cavities typically fall in the range of 5–30.^28–38^ Our result represents a record-high value for solution-processed perovskite thin-film devices.

Furthermore, spatially resolved PL mapping (Figure 5f) provides definitive evidence of the effective coupling between the ultrathin CsPbBr_3_ layer and the photonic membrane modes. At the intrinsic emission peak of the NCs (λ = 510 nm, bottom panel), the PL intensity indicates the square profile of the patterned hole-array region. This overall intensity lift arises from the freestanding nature of the membrane, which effectively suppresses substrate leakage channels compared to the surrounding solid substrate area. However, a different spatial distribution is observed at the resonance wavelength (λ = 520 nm, top panel). The emission exhibits a spatial contraction, becoming strongly localized close to the center of the patterned area. This central concentration is a characteristic signature of quasi-BIC resonances in finite-size photonic structures, where the resonant mode profile is modulated by the finite-size effect.^18,44^ This clear distinction confirms that the giant PL amplification originates from specific coupling to the degenerate quasi-BIC modes.

In conclusion, this work demonstrates an innovative and robust strategy for maximizing light-matter interaction by combining two critical advances. Structurally, the monolayer graphene interface functions as a necessary support, allowing for the realization of a continuous ultrathin (∼20 nm) active layer suspended over an air-hole photonic membrane. This morphology effectively eliminates capillary-induced leakage and scattering losses. Optically, this ultrathin geometry is essential for maximizing the energy localization from the dual-mode degenerate quasi-BICs. By strictly confining the emitters within the constructive field superposition of these orthogonal modes, we achieved a giant Purcell enhancement that is unattainable in thicker films. This integration of 2D-material scaffolding for high quantum-efficiency nanomaterials and dual-mode degenerate quasi-BICs supported by the freestanding photonic membrane paves the way for next-generation ultrathin light sources and high-performance nanophotonic devices.

## Supplementary Material



## Notes

Experimental procedures for the synthesis and transfer of CsPbBr_3_ nanocrystals and graphene films; details of optical characterization setups including micro-PL and time-resolved PL; structural characterizations via TEM and large-area SEM evaluating fabrication yield; optical experimental data including graphene control experiments, angle-resolved transmittance spectra, and power-dependent PL measurements; and detailed numerical simulation methodology (RCWA and FEM) with supporting calculations for band structures, multipolar field distributions, confinement factors, and Purcell factors (PDF)
