# Supplementary material for: Graphene-Scaffolded Ultrathin Perovskite Nanocrystal Films for Amplifying Energy Localization via Dual-Mode Nonhybridizing Quasi-BICs
Source: Nano Lett. 2026 Mar 28;26(13):4439–48. doi: 10.1021/acs.nanolett.6c00330 (PMC13067381; doi:10.1021/acs.nanolett.6c00330)
Supplement: Supplementary file 1 [file nl6c00330_si_001.pdf]

Supporting Information

Graphene-Scaffolded Ultrathin Perovskite Nanocrystal Films for Amplifying Energy Localization via Dual-Mode Non-Hybridizing quasi-BICs

Ya-Lun Ho[a*]*, Mu-Hsin Chen*[a]*, Tsung-Hsin Liu*[b,c]*, Fong-Liang Hsieh*[a,d]*, Chun-Hao Chiang*[a,d]*, Chih-Zong Deng*[a]*, Man-Hong Lai*[e]*, Jessie Shiue*[f,g]*, Shuaicheng Liu*[h]*, Haruyuki Sakurai*[i]*, Jui-Han Fu*[j]*, Kuniaki Konishi*[i]*, Vincent Tung*[j]*, Yu-Ming Chang*[e,k]*, Chun-Wei Chen*[b,d,e,k*] *and Shao-Ku Huang*[d*]

[a] Research Center for Electronic and Optical Materials, National Institute for Materials Science (NIMS), Ibaraki 305-0044, Japan

[b] International Graduate Program of Molecular Science and Technology (NTU-MST), National Taiwan University, Taipei 10617, Taiwan

[c] Molecular Science and Technology Program, Taiwan International Graduate Program (TIGP), Academia Sinica, Taipei 11520, Taiwan

[d] Department of Materials Science and Engineering, National Taiwan University, Taipei 10617, Taiwan

[e] Center for Condensed Matter Sciences, National Taiwan University, Taipei 10617, Taiwan

[f] Institute of Atomic and Molecular Science, Academia Sinica, Taipei 10617, Taiwan

[g] Institute of Physics, Academia Sinica, Taipei 11520, Taiwan

[h] Department of Physics, School of Science, The University of Tokyo, Tokyo 113-0033, Japan

[i] Institute for Photon Science and Technology, School of Science, The University of Tokyo, Tokyo 113-0033, Japan

[j] Department of Chemical System Engineering, School of Engineering, The University of Tokyo, Tokyo 113-8656, Japan

[k] Center of Atomic Initiative for New Materials (AI-MAT), National Taiwan University, Taipei 10617, Taiwan

1. Synthesis of All-Inorganic Perovskite $CsPbBr_3$ NCs

Lead (II) bromide ($PbBr_2$, 99%) and cesium carbonate (Cs_2CO_3, ReagentPlus, 99%) were obtained from Sigma-Aldrich. The solvents and ligands, including 1-octadecene (ODE, technical grade 90%), oleylamine (OAM, 95%), ethyl acetate (EA, 99.5%), and methyl ethyl ketone (MEK, 99%), were purchased from ACROS Organics. Anhydrous *n*-hexane (Hex, extra dry, water <50 ppm) stored with molecular sieves was also acquired from ACROS. Oleic acid (OA, laboratory reagent grade 70%) was supplied by Fisher Scientific. All reagents were used directly as received without any further purification.

The colloidal $CsPbBr_3$ NCs were synthesized following the hot-injection method described in the reference [1-3]. Initially, a cesium-oleate (Cs-oleate) precursor was prepared by loading Cs_2CO_3 (0.4 g), OA (1.2 mL), and ODE (15 mL) into a 50 mL three-necked flask. The mixture was degassed under vacuum at 120 °C for 1 h and subsequently heated to 150 °C under an argon atmosphere. The reaction proceeded for approximately 25 min until the solution turned clear, indicating the formation of Cs-oleate. For the perovskite synthesis, $PbBr_2$ (0.069 g) and ODE (5 mL) were loaded into a separate 25 mL three-necked flask and dried under vacuum at 120 °C for 1 h. The system was then switched to an argon atmosphere and heated to 130 °C. Ligands consisting of OA (0.5 mL) and OAM (0.5 mL) were injected to solubilize the $PbBr_2$. Once the lead salt was completely dissolved, the pre-heated Cs-oleate precursor (0.4 mL) was rapidly injected into the reaction vessel, followed by immediate cooling in an ice-water bath to quench the nucleation. The resulting $CsPbBr_3$ NCs were isolated and purified twice using a mixed solvent system of EA and MEK (volume ratio 1:9). The precipitate was collected by centrifugation at 4000 rpm and finally redispersed in anhydrous *n*-hexane to achieve a colloidal concentration of 5 mg/mL.

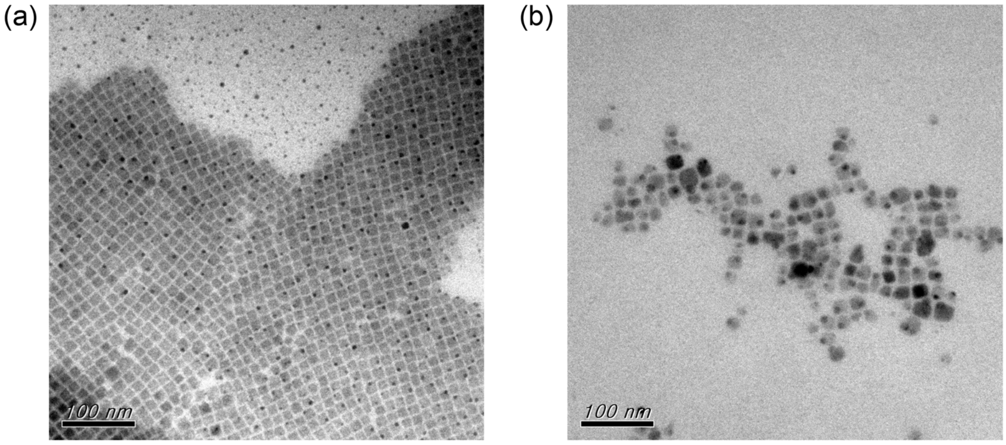

Figure S1. TEM characterization of the solution-processed $CsPbBr_3$ nanocrystals. (a) Low-magnification TEM image showing a densely packed and well-ordered superlattice of $CsPbBr_3$ NCs, demonstrating high structural consistency and well-defined cubic morphology with an average edge length consistent with high-quality perovskite emitters. **(b)** TEM image of more dispersed $CsPbBr_3$ NCs. Scale bars: 100 nm.

Reference

1. Protesescu, L.; Yakunin, S.; Bodnarchuk, M. I.; Krieg, F.; Caputo, R.; Hendon, C. H.; Yang, R. X.; Walsh, A.; Kovalenko, M. V. Nanocrystals of cesium lead halide perovskites ($CsPbX_3$, X = Cl, Br, and I): Novel optoelectronic materials showing bright emission with wide color gamut. *Nano Lett.* **2015**, *15*, 3692-3696.
2. Xing, D.; Lin, C. C.; Ho, Y. L.; Kamal, A. S. A.; Wang, I. T.; Chen, C. C.; Wen, C. Y.; Chen, C. W.; Delaunay, J. J. Self-healing lithographic patterning of perovskite nanocrystals for large-area single-mode laser array. *Adv. Funct. Mater.* **2020**, *31*, 2006283.

2. Synthesis and Transfer of Graphene Film

A graphene film (4 cm × 4 cm) was synthesized on Cu foil via a low-pressure chemical vapor deposition (LPCVD) process and subsequently transferred onto a planar SiNx TEM grid substrate using a poly(ethylene-vinyl acetate) (EVA)–assisted transfer method. EVA (5 wt% vinyl acetate in o-xylene) was spin-coated onto the graphene/Cu surface. After being cut into 2 mm × 2 mm pieces, the EVA/graphene/Cu stack was subjected to sequential etching in 2.0 M and 0.5 M $FeCl_3$ solutions to remove the Cu substrate. Following etching, the samples were rinsed three times with deionized (DI) water to remove residual $FeCl_3$. To mitigate graphene p-doping, a buffered oxide etchant (NH_4F:HF = 6:1) was applied, followed by three additional DI-water rinses to eliminate residual BOE. Before transferring the EVA/graphene film onto the SiNx TEM grid, the substrate was treated with oxygen plasma at 30 W for 5 minutes to remove surface contaminants and render the surface hydrophilic. After transfer, the sample was heated at 80 °C for 10 minutes and subsequently immersed in an o-xylene bath at 80 °C for 10 minutes to fully remove the EVA layer.

3. Scalability and fabrication yield of the graphene-scaffolded $CsPbBr_3$ NC film

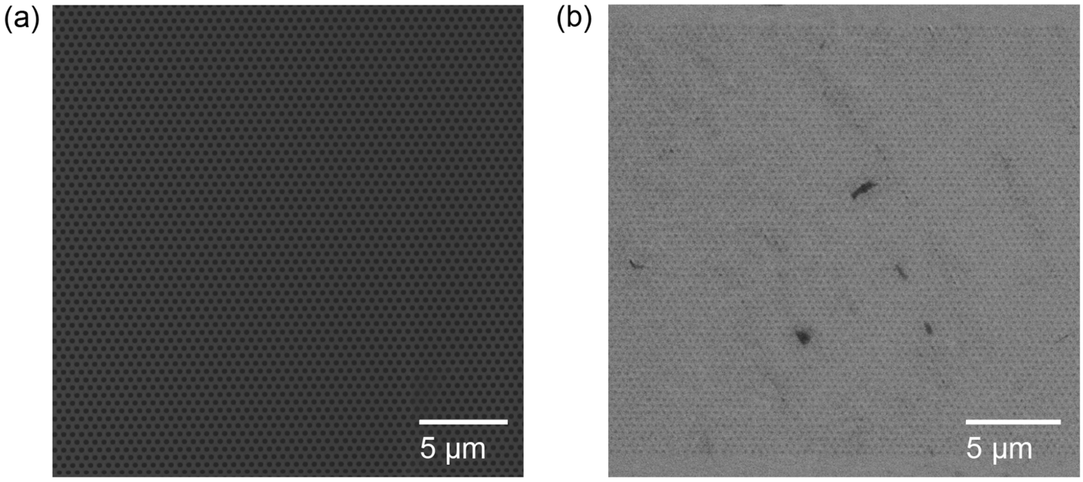

Figure S2. SEM characterization evaluating the structural yield of the graphene-scaffolded architecture. (a) SEM image demonstrating the high uniformity and defect-free nature of the monolayer graphene transferred onto the suspended air-hole photonic membrane under optimized conditions. (b) Top-view SEM image of the same architecture after the deposition of the 20-nm-thick CsPbBr3 NC film.

To assess the scalability and fabrication yield of our graphene-scaffolding strategy, we performed large-area SEM inspections (Figure S2). As shown in Figure S2a, the initial transfer of the monolayer graphene provides near-perfect coverage over the photonic membrane without any macroscopic defects. Following the deposition of the 20-nm thick $CsPbBr_3$ NC film (Figure S2b), statistical analysis encompassing over 4,500 air holes across 25 × 25 μm^2 devices confirms a remarkably high structural yield of >99% for the fully suspended ultrathin film.

4. Control experiment evaluating the optical influence of the graphene scaffold

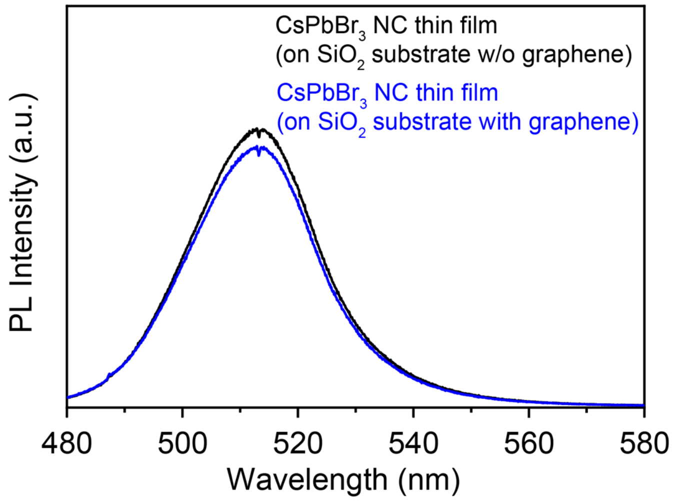

Figure S3. PL spectra of $CsPbBr_3$ NC thin films on planar SiO_2 substrates without (black curve) and with (blue curve) a monolayer graphene. The spectra show only a minor reduction (~7%) in PL intensity in the presence of graphene, indicating minimal non-radiative quenching or absorption losses.

5. Mode classification and experimental verification of quasi-BICs

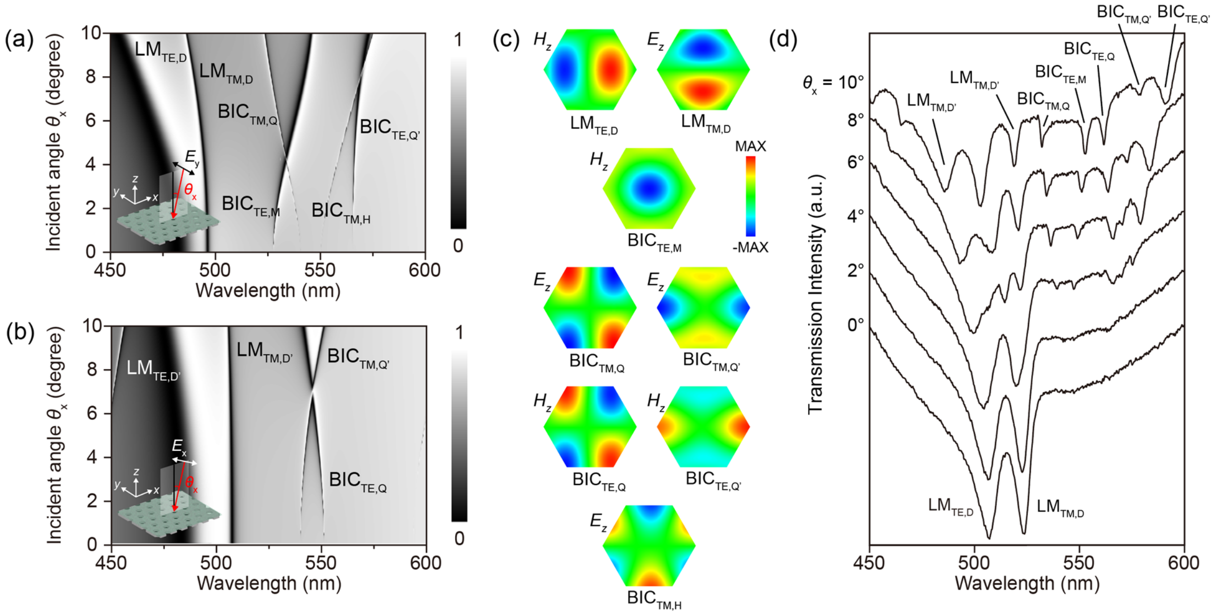

Figure S4. Simulated angle-resolved transmittance spectra of the photonic membrane under (a) y-polarized and (b) x-polarized excitation, mapped by varying the incident angle θ_x for a membrane thickness of T_{SiN} = 190 nm. The spectra map the dispersion of the resonances, wherein the LMs exhibit distinct splitting into multiple branches at larger incident angles. A slight spectral mismatch between the symmetry-protected degenerate modes at normal incidence (θ_x = 0°) is observed in the rigorous coupled-wave analysis (RCWA) calculations; this computational artifact originates from the discrete rectangular meshing and the different periodicities along the x- and y-directions. Exact eigenfrequency calculations confirming the modal degeneracy are provided in Figure S5. (c) Simulated out-of-plane field distributions (H_z or E_z) of the resonances indicated in (a) and (b), revealing their distinct TE- or TM-like multipolar field configurations. (d) Experimentally measured angle-resolved transmittance spectra with non-polarized light, validating the spectral positions and dispersion behaviors of the LMs and quasi-BICs.

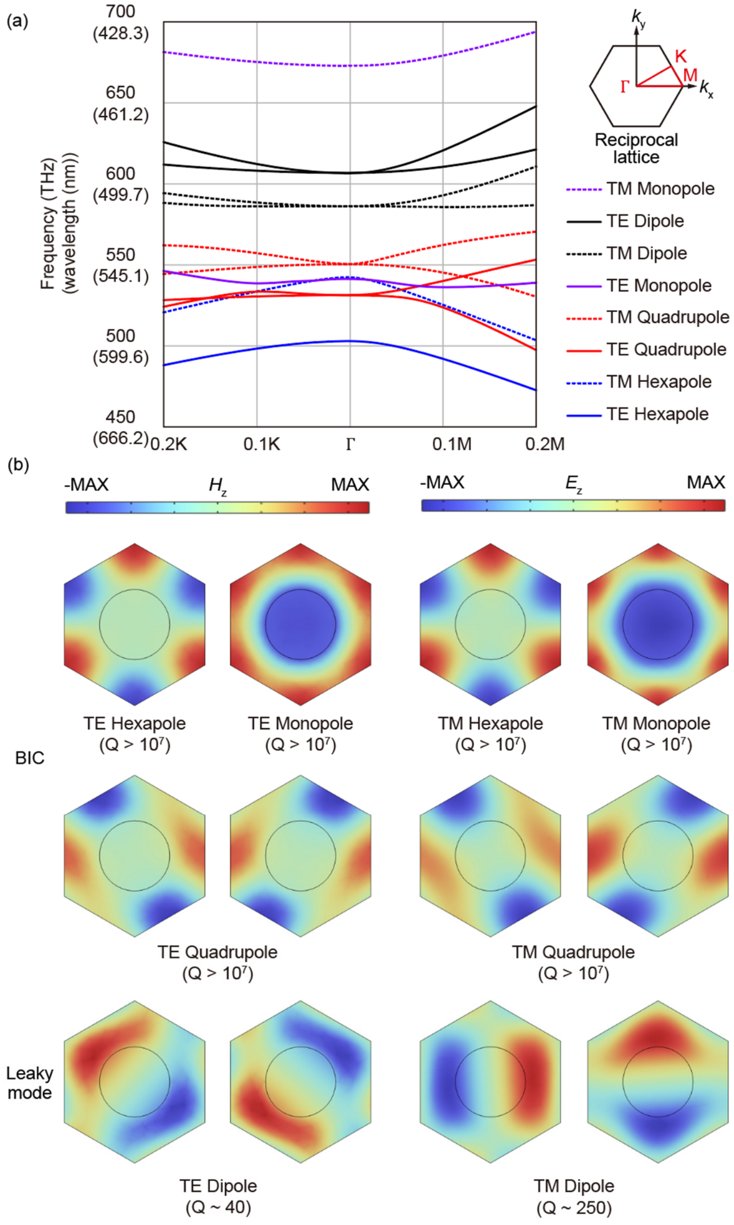

Figure S5. Dispersion and mode profiles of the photonic membrane. (a) Band structure of the photonic crystal membrane with a thickness T = 190 nm. The modes are labeled as TE or TM Mie resonances according to their multipole decomposition at the Γ point. (b) Simulated out-of-plane magnetic (H_z) and electric (E_z) field distributions for the TE and TM resonances at the Γ point, respectively. The associated Q-factors highlight the contrast between the non-radiative bound states in the continuum (BICs, $Q > 10^7$) and the radiative leaky modes.

6. Power-dependent PL measurements

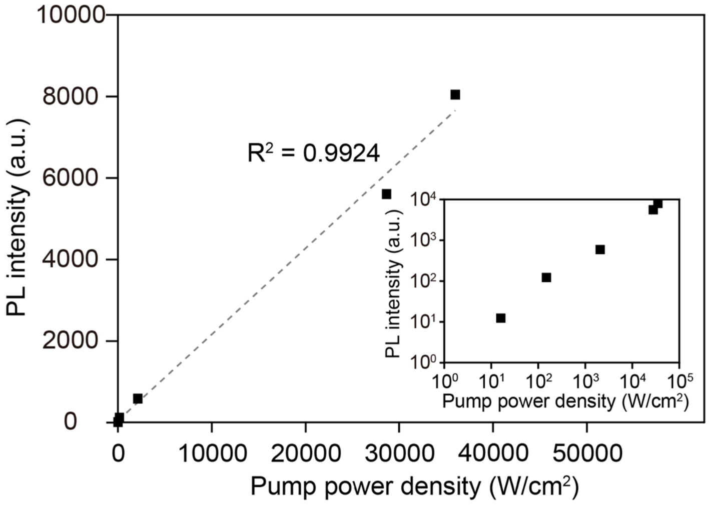

Figure S6. Power-dependent PL measurements of the graphene-scaffolded $CsPbBr_3$ NC film. The main panel shows the PL intensity as a function of the pump power density under a 405-nm continuous-wave laser excitation. The dashed line represents a linear fit to the experimental data (R^2 = 0.9924), demonstrating a linear power dependence. The inset displays the same experimental data plotted on a log-log scale. This linear behavior confirms the absence of optical saturation or photobleaching effects across the measured power range, validating that the PL enhancement factor evaluated at the standard operating condition (150 W/cm²) reflects the intrinsic amplification capability of the photonic membrane architecture.

7. Confinement factors of leaky modes and quasi-BICs

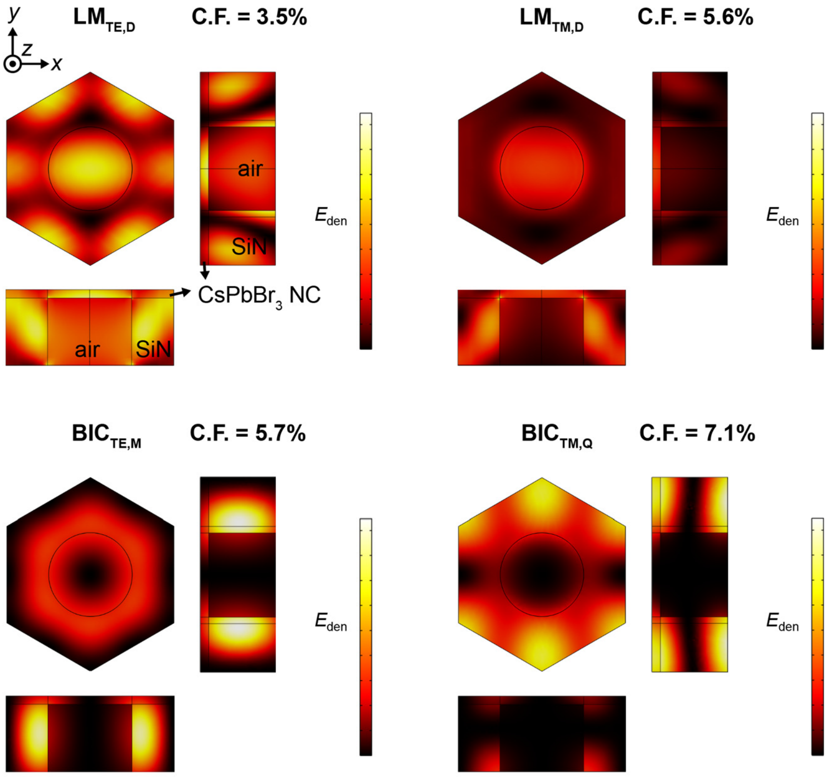

Figure S7. Simulated top-view and cross-sectional electric field energy density distributions for the leaky modes ($LM_{TE,D}$ and $LM_{TM,D}$) and the quasi-BICs ($BIC_{TE,M}$ and $BIC_{TM,Q}$). The calculated confinement factors (C.F.) within the 20 nm thick $CsPbBr_3$ NC film are indicated for each mode. The results reveal that the confinement factors for the quasi-BICs (5.7%–7.1%) are comparable to those of the leaky modes (3.5%–5.6%). This indicates that the spatial mode overlap alone cannot account for the giant PL amplification, further confirming that the enhancement is fundamentally driven by the amplified local density of optical states.

8. Numerical Simulations

The optical properties of the graphene-$CsPbBr_3$ photonic membrane were theoretically investigated using both rigorous coupled-wave analysis (RCWA) and finite element method (FEM) simulations. The far-field angle-resolved transmittance spectra and the dispersion relations of the resonance modes were calculated using the RCWA method (DiffractMOD, RSoft Design Group, USA). The electric field **E** is normalized by the electric field amplitude of the incident light. The electric energy density is defined as $U_E = ½\int \mathrm{Re}[\varepsilon(\mathbf{r}')]|\mathbf{E}|^2\, dV$, where **E** is the electric field, ε is the spatially dependent permittivity, and V is the volume of the simulation grid.

To quantify the near-field enhancement, FEM simulations were conducted using COMSOL Multiphysics (COMSOL, Inc., USA). The distributions of the electric field (E), magnetic field (H), and the Purcell factors, as well as the dispersion diagram were computed using the Eigenfrequency solver, while the radiation enhancement was analyzed using the Frequency Domain solver.

The details of Purcell factor calculation are described as follows. The maximum Purcell factor was calculated following the definition: [1][2]

$$F_{Purcell}^{max} = \frac{3}{4\pi^2}\left(\frac{\lambda}{n}\right)^3 \frac{Q}{V_{mode}}$$

in which λ is the free space wavelength, n is the refractive index of the gain material, Q is the Q-factor, and V_{mode} is the mode volume.

While V_{mode} is the effective volume of the electromagnetic energy considering distribution, such is mathematically defined as the ratio between the total electric energy and the maximum electric energy density:

$$V_{mode} = \frac{\iiint \varepsilon(r)|E(r)|^2 d^3r}{\max\left(\varepsilon(r)|E(r)|^2\right)}$$

in which ε is the permittivity of the gain material, and E is the electric field.

In the weak coupling regime, the spatial distribution of the Purcell factor follows the partial local density of states (LDOS). Consequently, the local Purcell factor can be mapped using the normalized electric field intensity of the fundamental cavity mode, expressed as: [3][4]

$$F_{Purcell}(r) = F_{Purcell}^{max} \frac{\varepsilon(r)|E(r)|^2}{\max\left(\varepsilon(r')|E(r')|^2\right)}$$

[1] Purcell, E. M.; Torrey, H. C.; Pound, R. V. Resonance Absorption by Nuclear Magnetic Moments in a Solid. *Phys. Rev.* **1946**, *69* (1-2), 37–38.

[2] Maier, S. A. Effective Mode Volume of Nanoscale Plasmon Cavities. *Opt. Quantum Electron.* **2006**, *38* (1), 257–267.

[3] Novotny, L.; Hecht, B. *Principles of Nano-Optics*, 2nd ed.; Cambridge University Press: Cambridge, U.K., 2012

[4] Machiya, H.; Yamashita, D.; Ishii, A.; Kato, Y. K. Evidence for Near-Unity Radiative Quantum Efficiency of Bright Excitons in Carbon Nanotubes from the Purcell Effect. *Phys. Rev. Res.* **2022**, *4* (2), L022011.

9. Optical Characterization

Micro-photoluminescence (μ-PL) measurements were performed at room temperature using a home-built confocal microscopy system. The samples were excited by a 405-nm continuous-wave diode laser focused through a 10× objective lens (NA = 0.25) to a diffraction-limited spot. The emission from the sample was collected by the same objective, passed through a long-pass filter to reject the excitation light, and coupled into a high-resolution spectrometer via a multimode optical fiber (core diameter = 105 μm). Spatially resolved PL mapping was acquired by raster-scanning the sample using a motorized piezoelectric stage. Time-resolved PL measurements were performed using time-correlated single photon counting system (PicoHarp 300, PicoQuant, Germany) with a 405-nm picosecond pulsed laser focused through a 10× objective lens (NA = 0.25) to a diffraction-limited spot.
